# Survey data on bullying involvement among school-going adolescents in India

**DOI:** 10.1016/j.dib.2024.110061

**Published:** 2024-01-14

**Authors:** Sruthi Suresh, R. Vijaya

**Affiliations:** Department of Psychology, CHRIST (Deemed to be University), Bengaluru, India

**Keywords:** Bullying perpetration, Bullying victimization, Traditional classrooms, Virtual classrooms, Middle school, High school

## Abstract

The data was collected from 169 school-going adolescents of grades sixth to twelfth from two cities in South India. The data set contains information of the frequency and type of bullying involvement (perpetration and victimization; physical, verbal, and social) among the participants in traditional and virtual classrooms. The data set can be used by all stakeholders to identify the frequency and types of bullying involvement among Indian adolescents. Further, relevant policies and programs can be developed that is tailored to the Indian adolescent population and the specific sociodemographic groups that are most vulnerable. Researchers can also replicate the study in various parts of India to compare bullying involvement among school-going adolescents across different parts of the nation.

Specifications Table

**Table:** 

Subject	Psychology; Developmental and Educational Psychology; Social and Personality Psychology
Specific subject area	The study of behaviour, specifically bullying involvement, in schools, grades six to twelfth, across the developmental category of early, middle, and late adolescence
Data format	Raw
Type of data	Table
Data collection	The data was collected using survey forms consisting of 51 questions. The first seven questions related to the sociodemographic profile of the participant. The participant's involvement in bullying in traditional classrooms (36 items) and virtual classrooms (8 items) was assessed using the remaining items. A purposive sample of school-going adolescents of grades sixth to twelfth were the respondents. A total of 169 responses were obtained from the participants after collecting informed consent form from the participants and obtaining the required parental permission.
Data source location	Institution: CHRIST (Deemed to be University) City/Town/Region: Bengaluru Country: India
Data accessibility	Repository name: Mendeley Data Data identification number: https://doi.org/10.17632/sxf9kkb6t2.3 Direct URL to data: https://data.mendeley.com/datasets/sxf9kkb6t2/3

## 1 Value of the Data

• The data enables researchers to examine involvement of school-going adolescents in bullying in both traditional and virtual classrooms in India.
• The data can be used to identify the sociodemographic groups (based on gender, age, and grade level) of school-going adolescents who are most vulnerable to being involved in bullying.
• The data allows researchers to examine different forms of bullying and victimization – physical, verbal, social/relational – and compare the occurrence of bullying across traditional and virtual classrooms.

## 2 Data Description

The participants were school-going adolescents of grades six to twelve in two cities, Chennai and Bengaluru, in South India. A post-hoc sample size calculation using G*Power indicated a power of 0.98 for 169 participants and an effect size of 0.3. A purposive sampling technique was used wherein the researcher reached out to schools that met the inclusion and exclusion criteria (Table 1) and offered to conduct a workshop on school bullying. Schools which were coeducational, English medium, and had conducted both traditional and virtual classrooms were invited to participate in the study. Adolescent students of grades six to twelve were the focus of the study as research has indicated that it is the group most vulnerable to school bullying [1,2]. A 30 to 45 min session was conducted for the students of the participating schools. The session was led by student responses and discussed the meaning, types, roles, and means of regulating bullying in schools. The workshop was used to present the definition of bullying to ensure the responses to the self-report questionnaires were based on an adequate understanding of the concept [3]. The audience of the sessions was invited to participate in the study and those who consented were provided with parental permission forms.

**Table 1:** Inclusion and exclusion criteria for selection of schools.

Inclusion Criteria	Exclusion Criteria
Schools should have grades 6 to 12, i.e., middle and high school students	Schools that have explicit anti-bullying regulations or committees specified in their school handbooks
It should be an English medium school	
The school should have conducted traditional/physical/face-to-face classes before the pandemic and virtual classes during the pandemic	

A total of 700 students participated in the workshop during the months of August and September 2022. After introducing the study, 432 students (68%) consented to participate and filled in the forms. The participants were requested to submit the parental permission forms within a period of one week. Despite repeated reminders given through the school, only 169 participants (24%) submitted the parental permission forms. The limited parental response could have been due to forgetfulness of the students or parents or lack of understanding of the nature and importance of their response, as the researcher did not have a direct interaction with the parents.

The current data set outlines bullying involvement among school-going adolescents of grades six to twelve from two cities in South India. It includes data from both male (52.1%) and female (47.9%) adolescents mostly from the middle socioeconomic status (93.5%). The data was categorized based on age ranges of early (10–12 years) (35.5%), middle (13–15 years) (49.1%), and late (16–18 years) (14.8%) adolescence as well as based on grade level of middle school (grade 6 to 8) (53.8%) and high school (grade 9 to 12) (46.2%) (Table 2). The categorization of data into these groups is important because of the different challenges and developmental tasks faced by each of these groups. It will further facilitate the understanding of bullying among specific groups thereby enabling directed intervention and support programs.

**Table 2:** Demographic characteristics of the participants.

Variable	Sub-category	N	%
Socioeconomic Status	Low	5	3.0
	Middle	158	93.5
	High	2	1.2
	No Response	4	2.4
Gender	Male	81	47.9
	Female	88	52.1
Age	Early Adolescence (10–12)	60	35.5
	Middle Adolescence (13–15)	83	49.1
	Late Adolescence (16–18)	25	14.8
	No Response	1	0.6
Grade Level	Middle School (6–8)	91	53.8
	High School (9–12)	78	46.2

## 3 Experimental Design, Materials and Methods

The survey forms were comprised of the demographic profile sheet, Adolescent Peer Relations Questionnaire [4], and Online Victimization Scale for Adolescents - General Online Victimization subscale [5]. The present study used self-report questions that have been tested on various populations as they are reported to be efficient means of assessing student's experiences of bullying [6]. Further, individual items were used to assess the occurrence of different forms of bullying and victimization to obtain detailed understanding of the nature of student experiences (Tables 3 and 4). The participant profile sheet was used to provide details of the study and collect the basic information of the participants. The first section of the sheet outlined details of the study such as title, researchers, duration, and requirements of participation. Ethical considerations were also provided in the sheet such as voluntary participation, the right to withdrawal, the requirement of parental consent, confidentiality, the risks and benefits involved, and details of data storage and usage. The researcher guided participants through the ethical considerations with the support of teachers and the counsellor to ensure that participants had a clear understanding of their rights and nature of participation. The second section of the sheet requested participant details such as name, gender, age, grade, and socioeconomic status. The questions were simple and straightforward such as “What is your gender?” and “Which grade/class do you study in?”. The students were encouraged to provide only their initials to ensure anonymity and the data collected was stored in a secure Cloud platform accessible only to the research team.

**Table 3:** Descriptives of the data collected.

Variable	Sub-category	Mean	S.D.	Observed Range	Possible Range
Traditional Bullying		26.3	7.91	18–63	18–108
	Verbal Bullying (vb)	9.75	3.32	6–23	6–36
	Social Bullying (sb)	8.12	2.63	6–18	6–36
	Physical Bullying (pb)	8.39	3.31	6–23	6–36
Traditional Victimization		32.1	12.7	18–90	18–108
	Verbal Victimization (vv)	12.3	5.85	6–35	6–36
	Social Victimization (sv)	10.1	4.78	6–35	6–36
	Physical Victimization (pv)	9.65	4.51	6–29	6–36
Online Victimization (ov)		10.6	4.19	8–35	8–48

**Table 4:** Relationship between variables (Spearman's rho).

Variable	vb	sb	pb	vv	sv	pv	ov
Sb	0.42***	–					
Pb	0.43***	0.41***	–				
Vv	0.39***	0.30***	0.20**	–			
Sv	0.41***	0.40***	0.15	0.56***	–		
Pv	0.31***	0.32***	0.47***	0.40***	0.27***	–	
Ov	0.20***	0.27***	0.29***	0.37***	0.29***	0.29***	–
Age	0.10	−0.09	−0.09	0.22**	0.05	−0.10	−0.00
Grade	0.09	−0.12	−0.13	0.20**	0.07	−0.11	−0.01

Note. *p<.05, **p<.01, ***p<.001; vb = Verbal Bullying, sb = Social Bullying, pb =Physical Bullying, vv = Verbal Victimization, sv = Social Victimization, pv = Physical Victimization, ov = Online Victimization.

The Adolescent Peer Relations Questionnaire by Parada (2000) consists of 36 items to assess bullying experiences in the past year of school life. It is used to measure bullying and victimization experiences across three dimensions that are physical, verbal, and social. Each of the six subscales in this questionnaire is measured with six items. The bullying scale included items such as “Teased them by saying things to them” (verbal), “Got my friends to turn against a student” (social) and “Pushed or shoved a student” (physical). The victimization scale included items such as “I was teased by students saying things to me” (verbal), “I was left out of activities on purpose” (social), and “I was pushed or shoved” (physical). Participants are required to respond on a six-point Likert scale with options including never (1), sometimes (2), once or twice a month (3), once a week (4), several times a week (5) and every day (6). The score can be calculated by summing the individual scores for bullying and victimization. The subscale scores can also be calculated for physical, verbal, and social bullying and victimization by totalling the respective item scores. Participants who score a total score of 18 or a score of six on a subscale are said to have not been involved in bullying or victimization. Previous studies have reported a Cronbach's alpha value ranging from 0.68 to 0.79 for different items in the scale while using it to assess bullying and victimization among Indian adolescents [7]. In the present study, the items indicate a Cronbach's coefficient of 0.83 for bullying perpetration and 0.88 for bullying victimization indicating good reliability.

The Online Victimization Scale for Adolescents by Tynes et al. (2010) consists of 21 items across four subscales, general online victimization, sexual online victimization, individual online racial discrimination, and vicarious online racial discrimination. The present data set used the General Online Victimization subscale which consists of eight items. Items included were “People have posted mean or rude things about me on the Internet” and “I have been embarrassed or humiliated online”. Participants are required to respond by choosing one of the six given options including never (1), once (2), a few times a year (3), a few times a month (4), a few times week (5) and everyday (6). The total score is calculated by summing the responses to all eight items. The occurrence and frequency of online victimization will be assessed based on the sum of the total scores in the given 8 questions. The scale has previously been used in racially diverse samples, and the original study reported a Cronbach's alpha of 0.84 for the general online victimization subscale. In the present study, the items indicate a Cronbach's coefficient of 0.78.

## Limitations

Almost 50% of the participants were middle adolescents in middle school, hence the results can be applied to this group. However, the population distribution of middle schoolers and high schoolers follow a similar pattern as in the sample, with the number of students enrolled in the school reducing as the grade increases.

Most of the participants in this study were of middle socioeconomic status and from a metropolitan area in South India; consequently, the findings primarily apply to this group. Future studies can assess any socioeconomic-, syllabus-, and region-wise differences in school bullying. Most schools included in the study had a school counselor who was available on campus either part-time or full-time; hence, student involvement of school bullying may have been regulated due to guidance sessions conducted. The nature and frequency of bullying in schools without a school counselor can be examined and compared with those with a school counselor.

Social desirability bias could have influenced the results due to the self-report nature of the measures used in the study. Future research could also include peer nominations as a supplementary method to identify students involved in bullying incidents [8]. The study is cross-sectional in nature and hence is limited in capturing the changing patterns of bullying involvement in students. Future longitudinal research can investigate the relationship between traditional and online bullying involvement at the school age and bullying at higher education institutions, places of employment, and violence/abuse in personal relationships.

## Ethics Statement

The relevant informed consent from the student participant and parental permission from the parents were obtained from the participants included in the study. The research was carried out in accordance with the Declaration of Helsinki, and was reviewed and approved by the Institutional Review Board at CHRIST (Deemed to be University) (CU: RCEC/00243/11/21).

## CRediT authorship contribution statement

**Sruthi Suresh:** Conceptualization, Methodology, Investigation, Data curation, Writing – original draft, Visualization. **R. Vijaya:** Conceptualization, Methodology, Resources, Writing – review & editing, Supervision.

## Data Availability

Survey Data on Bullying Involvement among School-going Adolescents in India (Original data) (Mendeley Data)
